# Creating Clean Air Spaces During Wildland Fire Smoke Episodes: Web Summit Summary

**DOI:** 10.3389/fpubh.2021.508971

**Published:** 2021-02-15

**Authors:** Gilliane Davison, Karoline K. Barkjohn, Gayle S. W. Hagler, Amara L. Holder, Sarah Coefield, Curtis Noonan, Beth Hassett-Sipple

**Affiliations:** ^1^Air and Energy National Research Program, Office of Research and Development, U.S. Environmental Protection Agency, Research Triangle Park, NC, United States; ^2^Oak Ridge Institute for Science Education Postdoctoral Fellow hosted by Center for Environmental Measurement and Modeling, Office of Research and Development, U.S. Environmental Protection Agency, Research Triangle Park, NC, United States; ^3^Center for Environmental Measurement and Modeling, Office of Research and Development, U.S. Environmental Protection Agency, Research Triangle Park, NC, United States; ^4^Missoula City-County Health Department, Missoula, MT, United States; ^5^Center for Population Health Research, University of Montana, Missoula, MT, United States

**Keywords:** smoke, wildfire, indoor air filtration, portable air purifier, particulate matter

## Abstract

Effective strategies to reduce indoor air pollutant concentrations during wildfire smoke events are critically needed. Worldwide, communities in areas prone to wildfires may suffer from annual smoke exposure events lasting from days to weeks. In addition, there are many areas of the world where high pollution events are common and where methods employed to reduce exposure to pollution may have relevance to wildfire smoke pollution episodes and vice versa. This article summarizes a recent virtual meeting held by the United States Environmental Protection Agency (EPA) to share research, experiences, and other information that can inform best practices for creating clean air spaces during wildland fire smoke events. The meeting included presentations on the public health impacts of wildland fire smoke; public health agencies' experiences and resilience efforts; and methods to improve indoor air quality, including the effectiveness of air filtration methods [e.g., building heating ventilation and air conditioning (HVAC) systems and portable, free-standing air filtration systems]. These presentations and related research indicate that filtration has been demonstrated to effectively improve indoor air quality during high ambient air pollution events; however, several research questions remain regarding the longevity and maintenance of filtration equipment during and after smoke events, effects on the pollution mixture, and degree to which adverse health effects are reduced.

## Introduction

Wildland fire smoke is a global public health issue. Many communities are exposed to smoke from wildland fires, both wild and prescribed burns, for days, weeks, or even months in a given year. As wildfire seasons have lengthened and grown in severity due to a combination of climate change (1) and accumulated fuels within the landscape, wildland fire smoke episodes have worsened. The number of individuals adversely impacted by these smoke events is growing as the wildland urban interface (WUI) expands and at-risk populations increase. Wildland fire smoke can infiltrate indoors, emphasizing the importance for enhancing the science to inform decisions for creating cleaner indoor air spaces where residents can seek refuge. Thus, EPA's Office of Research and Development sponsored a web summit to review the current state of knowledge regarding the effectiveness of strategies to improve indoor air quality during wildland fire events.

The web summit, *Clean Air Spaces: Indoor Air Filtration to Protect Public Health During Wildland Fire Smoke Episodes – What are the Knowns and Unknowns?* included 15 presentations offering insights from multiple levels of government and the scientific research community. This virtual meeting brought together experts (Table 1) from across the U.S. and Canada to share information and identify key knowledge gaps. Table 2 summarizes important resources discussed by the speakers. Although this web summit had a North American focus, many of the mitigation strategies and the challenges discussed here can translate to other parts of the world where wildfires are common. This article provides a summary of the information presented during this meeting while acknowledging that there exists more research on addressing wildfire smoke public health effects and other high pollution events in other parts of the world.

**Table 1:** Presenters and presentation titles.

**Presenter**	**Organization**	**Title**
Ryan Allen	Simon Fraser University	Portable Air Cleaners, Cardiovascular Health, and Fetal Growth: Results from Randomized Studies in Canada and Mongolia
Joe Beres	U.S. Department of State	Reducing Exposure to Air Pollution by Improving Indoor Air Quality
Michael Bergin	Duke University	Does Indoor Air Filtration Improve Health?
Terry Brennan^*^	Camroden Associates, Inc	Making a Safe Indoor Space During High Pollution Outdoor Events
Wayne Cascio	U.S. EPA	Wildfire Smoke and Public Health - Why is the EPA Concerned?
Rengie Chan	Lawrence Berkeley National Laboratory	Health Benefits and Cost Estimates of Filtration Interventions During 2003 Southern California Wildfires
Sarah Coefield	Missoula City-County Health Department	Mitigation Measures in Missoula County: A Look at Smoke-readiness in Missoula County, Montana
Julie Fox	Washington Department of Health	Challenges in Protecting Health in Wildfire Smoke Response in Washington: A Growing List of Questions
Sarah Henderson	British Columbia Center for Disease and Control	Wildfire Smoke is the Worst Kind of House Guest
Pete Lahm	U.S. Forest Service (USFS)	Interagency Wildland Fire Air Quality Response Program
Andrew Persily^*^	National Institute of Standards and Technology	ASHRAE Indoor Air Quality Standards and Wildfire Smoke Events
Kris Ray	Confederated Tribes of the Colville Reservation	Filters in Our Lives, Decreasing Our Exposure to Wildland Smoke
Jeffery Siegel	University of Toronto	Residential Forced Air Systems During Extreme Events: Help or Hindrance?
Jeff Wagner	California Department of Public Health (CDPH)	Potential Health Impacts of Particles and Gases Emitted by Wildfires
Jeffery Williams	California Air Resources Board (CARB)	Reducing Exposure to Wildfire Smoke

* *Presentations not publicly available*.

*Presentations are available online at https://www.epa.gov/air-research/web-summit-presentations-clean-air-spaces-indoor-air-filtration-protect-public-health*.

**Table 2:** Overview of on-line resources referenced by presenters.

**Title**	**Summary**
**Indoor Air**	
Indoor Air Quality Scientific Findings Resource Bank - Berkeley Lab https://iaqscience.lbl.gov/indoor-air-quality-iaq-scientific-findings	Educational resources on indoor air quality with relevant sections on building ventilation, indoor VOC impact, and benefits of improved indoor air quality
Guide to Air Cleaners in the Home, 2^nd^ Edition - U.S. EPA https://www.epa.gov/sites/production/files/2018-07/documents/guide_to_air_cleaners_in_the_home_2nd_edition.pdf	Consumer guide providing tips for selecting a portable air cleaner (PAC) or heating, ventilation and air conditioning (HVAC) filter to reduce indoor air pollutants in homes
Residential Air Cleaners: A Technical Summary, 3rd edition - U.S. EPA https://www.epa.gov/sites/production/files/2018-07/documents/residential_air_cleaners_-_a_technical_summary_3rd_edition.pdf	Technical guide focusing on residential air cleaning devices and other technologies and their effectiveness in removing indoor air pollutants
Air Cleaning Devices for the Home – CARB https://ww3.arb.ca.gov/research/indoor/acdsumm.pdf	Basics about air cleaning devices and answers to frequently asked questions when comparing residential air cleaning methods
California Certified Air Cleaning Devices – CARB https://ww2.arb.ca.gov/our-work/programs/air-cleaners-ozone-products/california-certified-air-cleaning-devices	List of air cleaning devices tested for electrical safety and meeting California's ozone emission concentration limit of 0.050 parts per million
Directory of Certified Room Air Cleaners - Association of Home Appliance Manufacturers (AHAM) https://ahamverifide.org/directory-of-air-cleaners/	Searchable directory of certified air cleaners verified through AHAM's Room Air Cleaner Certification Program
**General Information on Air Pollutants**	
AirNow - U.S. EPA https://airnow.gov/index.cfm?action=airnow.mapcenter&mapcenter=1	U.S. Air Quality Index (AQI) data summarized using maps to show current and forecasted air quality conditions including current conditions related to fires
Integrated Science Assessments (ISA) - U.S. EPA https://www.epa.gov/isa	Syntheses of the policy-relevant science to inform reviews of the national ambient air quality standards (NAAQS), including potential health and environmental effects associated with criteria pollutant exposures, including PM and ozone
State of Global Air 2019 – Health Effects Institute, Institute for Health Metrics and Evaluation https://www.stateofglobalair.org/	Interactive website summarizing global, regional, and country-specific data on air quality and health
**Wildland Fires**	
Wildfire Smoke: A Guide for Public Health Officials - U.S. EPA, CDC, USFS, CARB, California Office of Environmental Health Hazard Assessment, U.S. Department of Health and Human Services https://www.airnow.gov/wildfire-smoke-guide-publications/	Guide providing public health officials with information to prepare for smoke events and to communicate health risks and take measures to protect the public when smoke is present; including a number of fact sheets containing information on indoor air filtration, preparing for the fire season, protecting children and others from wildfire smoke and ash, and ways to reduce smoke exposure
Smoke-Ready Toolbox - U.S. EPA https://www.epa.gov/smoke-ready-toolbox-wildfires	List of resources focused sharing information about the risks of wildland fire exposures and actions to reduce smoke exposure
Home and Community Clean Air Shelters to protect public health during wildfire smoke events - British Columbia CDC http://www.bccdc.ca/resource-gallery/Documents/Guidelines%20and%20Forms/Guidelines%20and%20Manuals/Health-Environment/WFSG_EvidenceReview_CleanAirShelters_FINAL_v3_edstrs.pdf	Document reviewing key points, evidence gaps, and considerations of studies investigating PACs to reduce particulate matter (PM) concentrations in residential and community indoor spaces
Montana Wildfire Smoke – Climate Smart Missoula https://montanawildfiresmoke.org	Current air quality information, resources to educate the public about wildfire smoke-related health risks, and strategies for creating cleaner indoor air spaces
Air Pollution and School Activities WA DOH https://www.doh.wa.gov/Portals/1/Documents/Pubs/334-332.pdf	Guide used by Washington State to provide recommendations regarding school activities (recess, outdoor physical education, athletic practices and events) based on air quality conditions

Presenters listed in Table 1: Wayne Cascio, Sarah Henderson, and Jeff Wagner summarized the health impacts of wildland fire smoke. Sarah Coefield, Julie Fox, and Peter Lahm discussed approaches to reduce public exposure to smoke through community preparedness and response. Andrew Persily, Jeffery Williams, Kris Ray, Rengie Chan, Jeffrey Siegel, and Terry Brennan discussed improving indoor air quality during wildland fires and other high ambient air concentration periods. Michael Bergin, Ryan Allen, and Joseph Beres discussed improving indoor air quality during non-wildfire high pollution episodes.

## Health Impacts of Smoke Exposure

Primary pollutants emitted directly from wildland fires and secondary pollutants formed in the atmosphere include many pollutants of concern for public health, such as particulate matter (PM), carbon monoxide (CO), volatile organic compounds (VOCs), polycyclic aromatic hydrocarbons (PAHs), and ozone (O_3_). While fine particulate matter (PM_2.5_) and O_3_ are included in EPA's Air Quality Index (AQI) calculations used to communicate air quality information to the public, other pollutants including coarse PM (PM_10−2.5_), ultrafine particles, and toxic gases, are omitted. Ongoing research has aimed to characterize physical and chemical emissions from wildfires to inform decision-makers and reduce public exposure to wildfire smoke [Wagner, Henderson, Table 1, (2–6)]. Wildfires that occur in urban areas may burn building materials resulting in emissions that may include combustion products of petroleum, plastics, asbestos, fiberglass, lead, and other toxic metals (7). Panelists highlighted the need for improved assessment of the physical and chemical characteristics of wildland fire smoke and how these unique characteristics may impact human health.

Individuals living near and downwind from wildland fires may be affected by smoke exposures, with health impacts that vary by individual susceptibility and vulnerability, duration and concentration of smoke exposure, fire and smoke behavior, and fuel type. Wildland fire smoke exposure is linked to a wide range of health effects including eye and throat irritation, increased risk of respiratory infections, and exacerbation of existing lung diseases (8, 9). Recent studies also suggest a potential association between wildfire smoke exposures and cardiac morbidity and all-cause mortality [Cascio, Table 1, (9)]. A recent EPA study found an increase in emergency department visits related to heart attacks, strokes, and respiratory effects related to smoke exposure in California, especially among adults ages 65 and over (10). Several studies observed an association between ambient PM and adverse birth outcomes [Allen, Table 1, (11, 12)], although birth outcomes studies specific to wildland fire smoke exposure are limited (13–15).

At-risk populations, including individuals with preexisting cardiovascular (CVD) or respiratory disease, children, older adults (age 65 and above), pregnant women, and fetuses, now account for 27% of the U.S. population (16–18) and future trends estimate that 45% of the U.S. population will develop some form of CVD by 2035 (19). Respiratory symptoms related to wildfire smoke, including impacts in asthmatics and children, have been documented in a number of studies (20–22). Within older adult populations, there is evidence that wildland fire smoke-related health risks are higher for women and African-Americans (23). Recent EPA research observed the increased risk of PM_2.5_-related cardiopulmonary hospitalizations among older adults was similar during wildfire smoke and non-smoke events, while the risk of asthma-related hospitalizations was higher during smoke days (24).

## State of Technology to Improve Indoor Air

### Reduce Air Infiltration

Sealing and positively pressurizing buildings can prevent smoke infiltration. Typically, new buildings are better-sealed than older buildings and have lower air change rates. Regardless of building design, occupants can seal exterior doors, windows, and unused vents using towels, duct tape, plastic, and other readily available materials, and can strategically open and close interior doors to reduce infiltration (Siegel, Brennan, Table 1). Better sealing the building and increasing building intake above exhaust (using window units if the building is not already equipped to bring in fresh air) can positively pressurize the building, thereby reducing polluted air leaks. However, during cold climate conditions, care must be taken when positively pressurizing a building as the likelihood of serious moisture problems in the walls is increased. If the fresh air intake is reduced, it is important to monitor PM and VOC concentrations, which may increase due to indoor sources (25, 26).

### Filter Indoor Air

**Figure 1:**
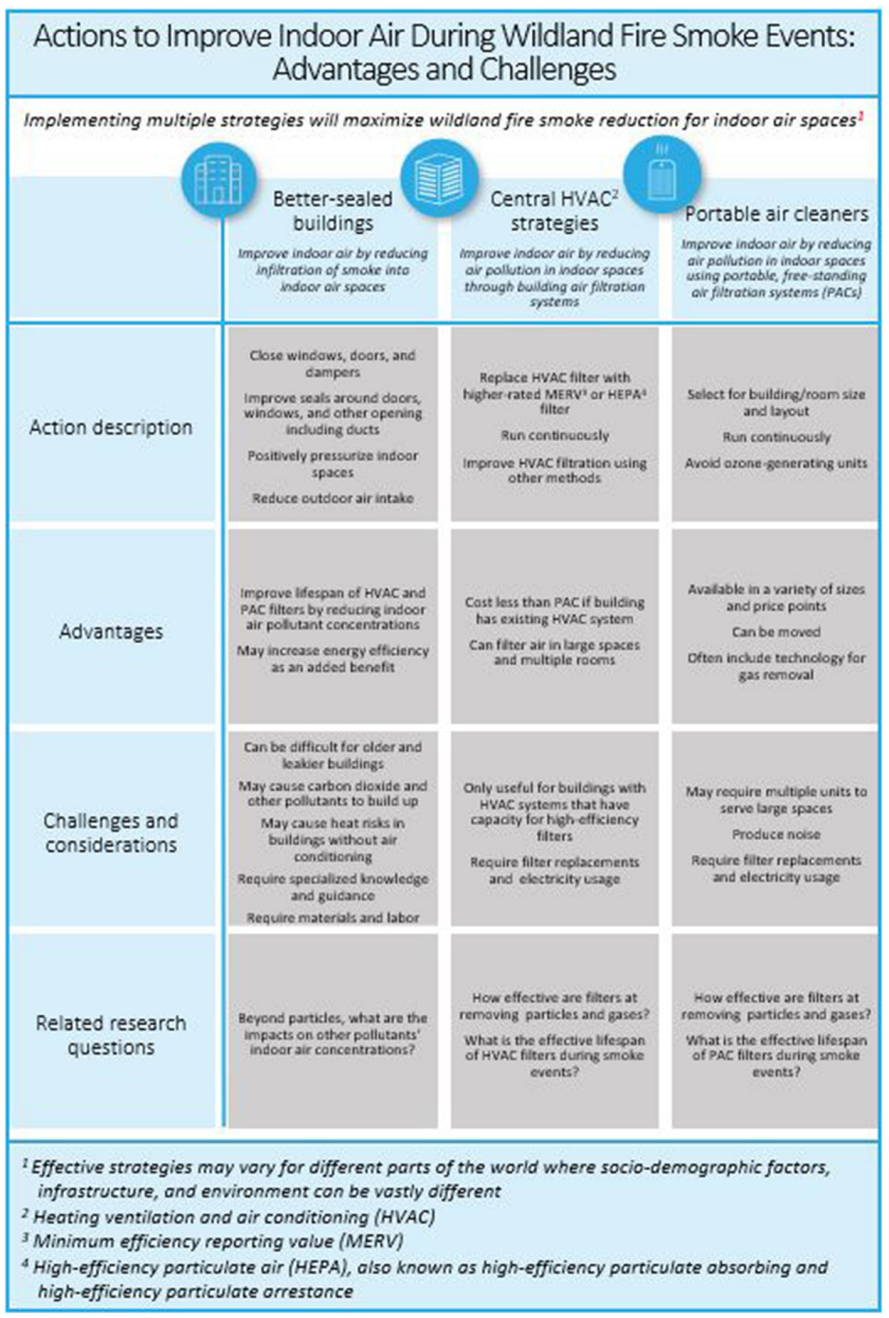
Actions to improve indoor air during wildland fire smoke events: advantages and challenges.

Mechanical and electronic filtration that does not produce ozone can effectively remove PM from indoor air (Brennan, Siegel, Williams, Table 1). Mechanical filters are described by their minimum efficiency reporting values (MERV) 1-16 and HEPA standards (ASHRAE 52.2) (27). The efficiency and the size range of PM removal varies with the MERV ratings with the smallest particles and highest removals achieved with HEPA filters and MERV 13 and greater. Unsurprisingly, studies have shown higher MERV filters are more effective at cleaning indoor air than lower MERV filters (28). The presenters recommended MERV 13 or greater filters for wildland fire smoke filtration, since these filters have some effectiveness removing the smallest particles (0.3–1.0 μm) that are most common in wildfire smoke. It is important to note that real-world removal efficiencies of mechanical filters are highly variable (29) due to user error, pressure drop across the filter as loading occurs that leads to filter bypass and flow reduction, manufacturer-reported filter lifetimes irrelevant for high concentration events, and filters' variable removal across particle sizes (Figure 1).

Currently, no national building guidelines or standards for wildland fire smoke-prone areas exist, though California has a new policy requiring improved filtration (MERV 13) for new construction (30). The American Society of Heating, Refrigerating and Air-Conditioning Engineers (ASHRAE) ventilation/indoor air quality and filtration standards (62.1, 62.2, 52.2, 170, 189.1, 189.3) do not address wildland fire smoke and have several limitations including the omittance of some critical operation and maintenance procedures. Additionally, the ASHRAE standards are not intended to provide protection for at-risk populations. Efforts to improve such standards may better prepare communities for wildland fire smoke events (Persily, Table 1).

Studies show that both HVAC filtration and PACs can significantly improve indoor air quality during wildland fire smoke events [Williams, Table 1, (31)]. HVAC systems operated based on a thermostat setting (i.e., operation triggered by temperature) filter air only while the fan is running, roughly 20 percent of the time (Siegel, Table 1). Residents may be more likely to run PACs continuously, though usage can be reduced due to maintenance issues, electricity cost, or noise levels, especially on high settings (Allen, Table 1). Unlike HVAC filters, PACs typically clean a single room and must be sized appropriately (Brennan, Table 1). Occupants can use a PAC in one room, move a PAC between rooms, or purchase PACs for multiple rooms. Due to the high cost of PACs (e.g., $100–$1,000, USD), residents sometimes choose lower-cost alternatives such as a box fan with an attached HVAC filter. This box fan system is being tested by multiple agencies and organizations for effectiveness and safety (Ray, Table 1). One limitation to air filtration is the need for electricity, which may not be available due to power outages or limited infrastructure. Under these conditions closing doors and windows during smoke events is effective at reducing indoor PM concentrations, although they should be opened back up after the air quality improves (32).

PM_2.5_ was the pollutant most frequently discussed during the web summit, but black carbon (BC), VOCs, and formaldehyde were also highlighted. Although filters can remove PM_2.5_ and BC, less is known about the effectiveness of these filtration methods to remove gaseous air pollutants in indoor air environments during wildfires (33–35). Furthermore, steps to reduce infiltration of ambient air into the indoor environment may increase the concentrations of pollutants associated with indoor air pollution sources.

### Health Benefits

Air filtration studies have primarily evaluated health outcomes during pollution events unrelated to wildland fire smoke. Results from Shanghai and Beijing (Bergin, Table 1), woodsmoke impacted homes, and Mongolia with indoor tobacco smoke indicate PACs reduce indoor PM_2.5_ concentrations and improve health markers for multiple at-risk populations including children, asthmatics, fetuses, and pregnant women (36–41). Moreover, reductions to indoor PM_2.5_ concentrations are robust to modest departures from full PAC usage compliance (42). While these studies may not draw direct comparisons to wildland fire events, they are instructive about the health benefits associated with reducing indoor PM_2.5_ exposures.

### Economic Benefits

The costs of wildland fire-related premature mortality and respiratory hospital admissions in the U.S. are estimated to be tens to hundreds of billions of dollars per year (43). Researchers examining a 2003 California wildfire found health-related economic benefits outweighed the cost for both a higher-rated MERV filter and the increased electricity cost of running PACs specifically in homes with older adults and the general population when used for multiple smoke events [Chan, Table 1, (15, 44)].

### Measure Indoor Air Quality

Indoor air pollutant measurements are necessary to understand potential health benefits from improving indoor air quality. Multiple speakers suggested that homeowners consider using low-cost sensors to check their indoor air quality. Low-cost sensors are useful to qualitatively monitor indoor and ambient air quality to assess relative changes and the impacts of indoor air pollution reduction strategies. In some cases, sensors are incorporated into air filtration devices to improve their performance and user engagement. Care must be taken to select sensors that perform adequately at high concentrations and wildfire relevant calibrations may be needed (45, 46).

## Efforts to Reduce Exposure Through Community Preparedness and Response

### Air Quality Response Program

The USFS Interagency Wildland Fire Air Quality Response Program was created to proactively prepare communities for managing the impacts of wildland fires on public health by predicting and mitigating wildland fire smoke exposure through modeling, monitoring, and messaging (Lahm, Table 1). The program uses fixed and temporary air quality monitoring equipment to better understand ambient air pollutant concentrations. Air Resource Advisors are dispatched to wildland fires to assist with understanding and predicting smoke impacts on the public and fire-fighting personnel. They analyze, summarize, and communicate these impacts to incident teams, air quality regulators, and the public.

### Community Preparedness

It is critical for the public to be educated about and understand actions they can take (such as sealing their homes and using filtration) to improve indoor air quality during smoke events. In addition to home environments, it is important to create cleaner classrooms, workplaces, and vehicle cabins, during wildland fire smoke events (Fox, Table 1). The strategies discussed here are specific for North America and different approaches may be needed in other parts of the world where infrastructure and building codes vary greatly.

Classrooms are a high priority because children are an at-risk population. Classrooms may present a challenge because they have high occupancy density and often lack adequate ventilation [Chan, Table 1, (25)].

Preparing workplaces for smoke events may require facilities, environmental health and safety, human resource departments, and managers working together to develop a smoke preparedness and response plan. During the 2018 Camp Fire, indoor PM_2.5_ in CARB's Sacramento office decreased when MERV 13 filters were replaced with MERV 16 filters and the office adopted better door control to limit smoke infiltration (Williams, Table 1).

Vehicle air filters are effective at reducing in-cabin PM_2.5_ concentrations, particularly if the cabin air is recirculated. However, it takes time to filter the air, so short trips or those with frequent stops where doors or windows are open, provide little protection. A case study in Uganda found that using a cabin air filter in recirculation mode decreases PM_2.5_ concentrations but increases CO_2_ concentrations significantly. A cabin air filter in pass-through mode reduces PM_2.5_ concentrations, though not as effectively, while only modestly raising CO_2_ concentrations (Beres, Table 1).

### Case Studies of Community Preparedness

In 2017, Missoula County, Montana experienced a record-breaking year for wildfire smoke. Following that wildfire season, MCCHD took measures to heighten community preparedness for future wildfire smoke events. MCCHD provided readily available smoke forecasts during wildfire smoke events, enhanced community awareness of smoke-related impacts, and provided approaches to improve indoor air quality. In 2017, MCCHD partnered with Climate Smart Missoula (CSM), a local nonprofit organization, to provide PACs to at-risk community members. In 2018, this partnership provided filtered air to more than 500 children by dispensing PACs to local daycares and preschools. Moving forward, MCCHD has secured commitments from new and remodeled schools in Missoula County to use HVAC filters with a minimum efficiency reporting value (MERV) of at least 13 during wildfire smoke events. In response to growing concerns about wildfire smoke events, the Montana Department of Public Health and Human Services has proposed changes to the Administrative Rules of Montana as they pertain to healthy learning environments in public schools to require a switch from HVAC filters with MERV 8 (no rated effectiveness for 0.3–1.0 μm) to MERV 13 (with at least 50% reduction for the 0.3–1.0 μm size range) (Coefield, Table 1).

The Washington Wildfire Smoke Impacts Advisory Group, comprised of state, local, tribal, and academic professionals, developed technical guidance and fact-based materials focused on communication, school and outdoor event closures, and air sensor measurements. Templates keep communication materials consistent but adaptable for location and audience. School cancellation recommendations in Washington are discussed in the WA Comprehensive Emergency Management Plan and are based on the *Washington Air Quality Advisory Guidance for Public Health Actions*, the expected length of the smoke episode, and driving visibility. Schools across Washington have varying HVAC systems; therefore, solutions must be tailored to each school depending on the air filtration systems available. These efforts highlight the need to balance scientific evidence, social infrastructure, and political will to create realistic and beneficial guidance and public policy^1^.

In both Washington and Montana, summertime heat is a concern. Many homes and public buildings do not have air conditioning and rely on open windows to cool indoor spaces. Closing windows to reduce infiltration of wildland fire smoke indoors may increase indoor temperatures and the risk of heat exhaustion, heat stroke, and premature death, especially in at-risk populations. Many residents must choose between risks associated with heat and risks from smoke exposure. The MCCHD recommends that residents who can tolerate some smoke exposure open their windows at night to let the cooler air inside and, once they have closed their windows, use PACs with true HEPA filters to reduce indoor PM_2.5_ concentrations. Residents who cannot tolerate smoke and do not have air conditioning may need to relocate.

## Conclusions and Remaining Research Questions

Wildland fires are a growing public health challenge within the US and globally, particularly as their magnitude and frequency are expected to continue to increase in the coming years due to the changing climate. The smoke from these fires is associated with adverse health effects and may be especially harmful to at-risk populations. Extensive research on indoor air quality and the experiences in areas with high ambient air pollution provide a basis for strategies to reduce the impact of wildland fire smoke. These strategies may vary for different parts of the world where socio-demographic factors, infrastructure, and environment can be vastly different despite the similar air quality challenges posed by wildfires. The expert panel brought together for this web summit highlighted that, while much information is known about the impacts of smoke from wildland fires and strategies to improve indoor air quality, substantial knowledge gaps remain. Collectively, the panelists raised several research questions regarding wildland fire smoke. Those directly related to indoor air exposures during wildland fire smoke events include the following:

- How do environmental and socio-demographic factors affect a community's susceptibility to health effects associated with wildland fire smoke?
- What are the best practices for addressing wildfire smoke-related indoor air quality impacts on communities with diverse infrastructure?
- What is the best way to communicate the health effects of smoke exposure to communities who have grown accustomed to smoke?
- What steps are individuals willing to take to protect their health and how do these actions differ based on socioeconomic status?
- How does combustion of different types of biomass and structural materials impact air pollutant mixtures, including particle size, and affect smoke infiltration indoors?
- How do health impacts vary based on the concentrations and length of smoke exposure?
  ◦ What are the effects of exposure to high concentrations of smoke (both particles and gases) lasting a few hours compared to lower concentrations lasting weeks or months?
  ◦ What effects are associated with repeated high concentration, short-term exposures that occur over multiple months and/or years?
  ◦ What intervention strategies are effective and practical to reduce short-term exposures for those at highest-risk and long-term exposures for all?
- What are the impacts of high temperatures and smoke impacts, especially in areas where air conditioning is limited?
- What are the best practices that homeowners or building operators can follow for improving indoor air quality during wildland fires?
- How can interventions such as PACs, clean air shelters and spaces, closures, and evacuations best be used?
- If air filtration devices are used:
  ◦ How does the effectiveness of these devices vary (e.g., lower-cost and lower-efficiency filtration options)?
  ◦ What is the effectiveness and lifespan of different filters and filter configurations for improving indoor air quality during wildfire smoke conditions; under what operating conditions?
- Without building HVAC systems or PACs, are there benefits for staying indoors during extended wildland fire events?
- Can low-cost air quality sensors or “smart” air cleaners with integrated sensors be used to evaluate and inform steps to improve indoor air quality during wildland fires?

Webinar participants strongly supported opportunities to continue dialogue, enhance coordination and collaboration, and leverage on-going and future research efforts to expand our understanding of ways to reduce exposures to wildland fire smoke by improving indoor air quality during such events.

## Author Contributions

GD, KB, AH, and BH-S developed primary text, including table and figure. GH, SC, and CN provided substantive edits. All authors contributed to the article and approved the submitted version.

## Conflict of Interest

The authors declare that the research was conducted in the absence of any commercial or financial relationships that could be construed as a potential conflict of interest.

## Footnotes

^1^Washington State Department of Health Smoke Toolkit (https://www.doh.wa.gov/CommunityandEnvironment/AirQuality/SmokeFromFires/SmokefromFiresToolkits) Last accessed, 10/15/2019.

## References

[1] Fourth National Climate Assessment (NCA4). Figure 25.4. (2018). Available online at: https://www.globalchange.gov/nca4 (accessed May 21, 2020).

[2] Adachi K Buseck PR. Atmospheric tar balls from biomass burning in Mexico. J Geophys Res Atmos. (2011) 116:D05204. 10.1029/2010JD015102

[3] Adar SD Filigrana PA Clements N Peel JL. Ambient Coarse particulate matter and human health: a systematic review and meta-analysis. Curr Environ Health Rep. (2014) 1:258–74. 10.1007/s40572-014-0022-z 25152864 PMC4129238

[4] CARB. Air Quality and the Wildland Fires of South California. (2003). Available online at: https://haze.airfire.org/webaccess/susan/HAQAST/Wildfires_TT/References/HealthImpacts/ARB_socal_report_revised_sept2010.pdf (accessed May 21, 2020).

[5] Kim YH Tong H Daniels M Boykin E Krantz QT McGee J. Cardiopulmonary toxicity of peat wildfire particulate matter and the predictive utility of precision cut lung slices. Part Fibre Toxicol. (2014) 11:29. 10.1186/1743-8977-11-29 24934158 PMC4072480

[6] Wolf RE Hoefen TM Hageman PL Morman SA Plumlee GS. Speciation of Arsenic, Selenium, and Chromium in Wildfire Impacted Soils and Ashes (2010-1242). (2010). Available online at: http://pubs.er.usgs.gov/publication/ofr20101242 (accessed May 21, 2020).

[7] Wagner J Ghosal S Whitehead T Metayer C. Morphology, spatial distribution, and concentration of flame retardants in consumer products and environmental dusts using scanning electron microscopy and Raman micro-spectroscopy. Environ Int. (2013) 59:16–26. 10.1016/j.envint.2013.05.003 23739093 PMC3759544

[8] Liu JC Mickley LJ Sulprizio MP Dominici F Yue X Ebisu K. Particulate air pollution from wildfires in the Western US under climate change. Clim Change. (2016) 138:655–66. 10.1007/s10584-016-1762-6 28642628 PMC5476308

[9] Reid CE Brauer M Johnston FH Jerrett M Balmes JR Elliott CT. Critical review of health impacts of wildfire smoke exposure. Environ Health Perspect. (2016) 124:1334–43. 10.1289/ehp.1409277 27082891 PMC5010409

[10] Wettstein ZS Hoshiko S Fahimi J Harrison RJ Cascio WE Rappold AG. Cardiovascular and cerebrovascular emergency department visits associated with wildfire smoke exposure in California in 2015. J Am Heart Assoc. (2018) 7:e007492. 10.1161/JAHA.117.007492 29643111 PMC6015400

[11] Li X Huang S Jiao A Yang X Yun J Wang Y. Association between ambient fine particulate matter and preterm birth or term low birth weight: an updated systematic review and meta-analysis. Environ Pollut. (2017) 227:596–605. 10.1016/j.envpol.2017.03.055 28457735

[12] Melody SM Ford J Wills K Venn A Johnston FH. Maternal exposure to short-to medium-term outdoor air pollution and obstetric and neonatal outcomes: a systematic review. Environ Pollut. (2019) 244:915–25. 10.1016/j.envpol.2018.10.086 30469286

[13] O'Donnell MH Behie AM. Effects of wildfire disaster exposure on male birth weight in an Australian population. Evol Med Public Health. (2015) 2015:344–54. 10.1093/emph/eov027 26574560 PMC4697771

[14] Holstius DM Reid CE Jesdale BM Morello-Frosch R. Birth weight following pregnancy during the 2003 Southern California wildfires. Environ Health Perspect. (2012) 120:1340–5. 10.1289/ehp.1104515 22645279 PMC3440113

[15] Wu JM Winer AJ Delfino R. Exposure assessment of particulate matter air pollution before, during, and after the 2003 Southern California wildfires. Atmos Environ. (2006) 40:3333–48. 10.1016/j.atmosenv.2006.01.056

[16] Cascio WE. Wildland fire smoke and human health. Sci Total Environ. (2018) 624:586–95. 10.1016/j.scitotenv.2017.12.086 29272827 PMC6697173

[17] Xu J Murphy SL Kochanek KE. Mortality in the United States, 2015. Hyattsville, MD: U.S. Department of Health and Human Services, Centers for Disease Control and Prevention, National Center for Health Statistics (2015). Available online at: https://www.cdc.gov/nchs/data/databriefs/db267.pdf

[18] Rappold AG Reyes J Pouliot G Cascio WE Diaz-Sanchez D. Community vulnerability to health impacts of wildland fire smoke exposure. Environ Sci Technol. (2017) 51:6674–82. 10.1021/acs.est.6b06200 28493694 PMC6372951

[19] American Heart Association. Cardiovascular Disease: A Costly Burden for America Projections through 2035. (2017). Available online at: https://healthmetrics.heart.org/wp-content/uploads/2017/10/Cardiovascular-Disease-A-Costly-Burden.pdf (accessed May 21, 2020).

[20] Arriagada NA Horsley JA Palmer AJ Morgan GG Tham R Johnston FH. Association between fire smoke fine particulate matter and asthma-related outcomes: systematic review and meta-analysis. Environ Res. (2019) 179 (Pt A):108777. 10.1016/j.envres.2019.108777 31593836

[21] Mirabelli MC Künzli N Avol E Gilliland FD Gauderman WJ McConnell R. Respiratory symptoms following wildfire smoke exposure: airway size as a susceptibility factor. Epidemiology. (2009) 20:451–9. 10.1097/EDE.0b013e31819d128d 19276978 PMC4517186

[22] Kunzli N Avol E Gauderman WJ Rappaport E Millstein J Bennion J. Health effects of the 2003 Southern California wildfires on children. Am J Respir Crit Care Med. (2006) 174:1221–8. 10.1164/rccm.200604-519OC 16946126 PMC2648104

[23] Liu JC Wilson A Mickley LJ Dominici F Ebisu K Wang Y. Wildfire-specific fine particulate matter and risk of hospital admissions in urban and rural counties. Epidemiology. (2017) 28:77–85. 10.1097/EDE.0000000000000556 27648592 PMC5130603

[24] DeFlorio-Barker S Crooks J Reyes J Rappold AG. Cardiopulmonary effects of fine particulate matter exposure among older adults, during wildfire and non-wildfire periods, in the United States 2008. Environ Health Perspect. (2019) 127:037006. 10.1289/EHP3860 30875246 PMC6768318

[25] Kaduwela AP Kaduwela AP Jrade E Brusseau M Morris S Morris J. Development of a low-cost air sensor package and indoor air quality monitoring in a California middle school: detection of a distant wildfire. J Air Waste Manag Assoc. (2019) 69:1015–22. 10.1080/10962247.2019.1629362 31199717

[26] Satish U Mendell MJ Shekhar K Hotchi T Sullivan D Streufert S. Is CO_2_ an indoor pollutant? direct effects of low-to-moderate CO_2_ concentrations on human decision-making performance. Environ Health Perspect. (2012) 120:1671–7. 10.1289/ehp.1104789 23008272 PMC3548274

[27] ASHRAE 52.2 Method of Testing General Ventilation Air-Cleaning Devices for Removal Efficiency by Particle size. Atlanta, GA: ASHRAE (2017).

[28] Singer BC Delp WW Black DR Destaillats H Walker IS. Reducing In-Home Exposure to Air Pollution. (2016). Available online at: https://ww3.arb.ca.gov/research/apr/past/11-311.pdf (accessed May 21, 2020).

[29] Azimi P Zhao D Stephens B. Modeling the impact of residential HVAC filtration on indoor particles of outdoor origin (RP-1691). Sci Technol Built Environ. (2016) 22:431–62. 10.1080/23744731.2016.1163239

[30] California Energy Commission. 2019 Building Energy Efficiency Standards for Residential and Nonresidential Buildings. (2019). Available online at: https://www.energy.ca.gov/programs-and-topics/programs/building-energy-efficiency-standards/2019-building-energy-efficiency (accessed May 21, 2020).

[31] Barn PK Elliott CT Allen RW Kosatsky T Rideout K Henderson SB. Portable air cleaners should be at the forefront of the public health response to landscape fire smoke. Environ Health. (2016) 15:116. 10.1186/s12940-016-0198-9 27887618 PMC5124284

[32] Reisen F Powell JC Dennekamp M Johnston FH Wheeler AJ. Is remaining indoors an effective way of reducing exposure to fine particulate matter during biomass burning events? J Air Waste Manag Assoc. (2019) 69:611–22. 10.1080/10962247.2019.1567623 30624153

[33] Barn P Larson T Noullett M Kennedy S Copes R Brauer M. Infiltration of forest fire and residential wood smoke: an evaluation of air cleaner effectiveness. J Expo Sci Environ Epidemiol. (2008) 18:503–11. 10.1038/sj.jes.7500640 18059421

[34] Fisk WJ Chan WR. Health benefits and costs of filtration interventions that reduce indoor exposure to PM2.5 during wildfires. Indoor Air. (2017) 27:91–204. 10.1111/ina.12285 26843218

[35] Henderson DE Milford JB Miller SL. Prescribed burns and wildfires in Colorado: impacts of mitigation measures on indoor air particulate matter. J Air Waste Manag Assoc. (2005) 55:1516–26. 10.1080/10473289.2005.10464746 16295277

[36] Cui X Li Z Teng Y Barkjohn KK Norri CL Fang L. Association between bedroom particulate matter filtration and changes in airway pathophysiology in children with asthma. J Amer Med Assoc Pediatr. (2020) 174:533–42. 10.1001/jamapediatrics.2020.0140 32250418 PMC7136863

[37] He L Li Z Teng Y Cui X Barkjohn K Norris C. Associations of personal exposure to air pollutants with airway mechanics in children with asthma. Environ Int. (2020) 138:105647. 10.1016/j.envint.2020.105647 32172043

[38] Barkjohn KK Bergin MH Norris C Schauer JJ Zhang Y Black M. Using low-cost sensors to quantify the effects of air filtration on indoor and personal exposure relevant PM_2.5_ concentrations in Beijing, China. Aerosol Air Qual Res. (2020) 20:297–313. 10.4209/aaqr.2018.11.0394

[39] Noonan CW Semmens EO Smith P Harrar SW Montrose L Weiler E. Randomized trial of interventions to improve childhood asthma in homes with wood-burning stoves. Environ Health Perspect. (2017) 125:097010. 10.1289/EHP849 28935614 PMC5915210

[40] Allen RW Carlsten C Karlen B Leckie S van Eeden S Vedal S. An air filter intervention study of endothelial function among healthy adults in a woodsmoke-impacted community. Am J Respir Crit Care Med. (2011) 183:1222–30. 10.1164/rccm.201010-1572OC 21257787

[41] Barn P Gombojav E Ochir C Laagan B Beejin B Naidan G. The effect of portable HEPA filter air cleaners on indoor PM_2.5_ concentrations and second hand tobacco smoke exposure among pregnant women in Ulaanbaatar, Mongolia: The UGAAR randomized controlled trial. Sci Total Environ. (2018) 615:1379–89. 10.1016/j.scitotenv.2017.09.291 29751442

[42] Ward TJ Semmens EO Weiler E Harrar S Noonan CW. Efficacy of interventions targeting household air pollution from residential wood stoves. J Expos Sci Environ Epidemiol. (2017) 27:64–71. 10.1038/jes.2015.73 26555475 PMC6384090

[43] Fann N Alman B Broome RA Morgan GG Johnston FH Pouliot G. The health impacts and economic value of wildland fire episodes in the U.S.: 2008–2012. Sci Total Environ. (2018) 610–611:802–9. 10.1016/j.scitotenv.2017.08.024 28826118 PMC6117838

[44] Delfino RJ Brummel S Wu J Stern H Ostro B Lipsett M. The relationship of respiratory and cardiovascular hospital admissions to the southern California wildfires of 2003. Occup Environ Med. (2009) 66:189–97. 10.1136/oem.2008.041376 19017694 PMC4176821

[45] Mehadi A Moosmüller H Campbell DE Ham W Schweizer D Tarnay L. Laboratory and field evaluation of real-time and near real-time PM_2.5_ smoke monitors. J Air Waste Manag Assoc. (2019) 70:158–79. 10.1080/10962247.2019.1654036 31403397

[46] Zheng T Bergin MH Johnson KK Tripathi SN Shirodkar S Landis MS. Field evaluation of low-cost particulate matter sensors in high- and low-concentration environments. Atmos Meas Tech. (2018) 11:4823–46. 10.5194/amt-11-4823-2018

